# Calixarenes: Generalities and Their Role in Improving the Solubility, Biocompatibility, Stability, Bioavailability, Detection, and Transport of Biomolecules

**DOI:** 10.3390/biom9030090

**Published:** 2019-03-05

**Authors:** Edilma Sanabria Español, Mauricio Maldonado Villamil

**Affiliations:** 1 Grupo GICRIM, Programa de Investigación Criminal, Universidad Manuela Beltrán, Avenida Circunvalar No. 60-00, Bogotá 111321, Colombia; 2 Departamento de Química, Facultad de Ciencias, Universidad Nacional de Colombia, Sede Bogotá, Cr. 30 No. 45-03, Bogotá 111071, Colombia

**Keywords:** calixarenes, complexion, amphiphilic assemblies, micelles, vesicles, liposomes, nanoparticles, encapsulation, transport of drugs, bioavailability, solubility of the drugs

## Abstract

The properties and characteristics of calix[*n*]arenes are described, as well as their capacity to form amphiphilic assemblies by means of the design of synthetic macrocycles with a hydrophilic head and a hydrophobic tail. Their interaction with various substances of interest in pharmacy, engineering, and medicine is also described. In particular, the role of the calix[*n*]arenes in the detection of dopamine, the design of vesicles and liposomes employed in the manufacture of systems of controlled release drugs used in the treatment of cancer, and their role in improving the solubility of testosterone and anthelmintic drugs and the biocompatibility of biomaterials useful for the manufacture of synthetic organs is emphasized. The versatility of these macrocycles, able to vary in size, shape, functional groups, and hydrophobicity and to recognize various biomolecules and molecules with biological activity without causing cytotoxicity is highlighted.

## 1. Introduction

In the fields of pharmacy, medicine, and nutrition, there are several macrocycle molecules that have been used for improving the solubility of the active ingredient of drugs and their stability and bioavailability for the determination of proteins and nucleic acids. They have even exhibited biological activity or value in sensitization therapies, as seen for example with cucurbit[*n*]urils [1], macrocyclic tetrapyrazolics [2], resorcinarenes [3], crown ethers [4], cyclodextrins [5], phthalocyanines [6], and calixarenes [7], among others. All of this has been possible due to the fact that these compounds are able to form a complex with molecules of biological interest such as antibiotics [1], proteins and nucleic acids [4], food nutraceuticals [5], and cholesterol [7], among many other compounds. In particular, the calixarenes are able to form a guest–host inclusion complex with molecules such as anthelmintics [8], testosterone [9], steroid hormones [10], antibacterial drugs [11], sugars [12], alditols [13], lectin [14], protein [15], and pesticides [16,17]. In addition, they have exhibited antiviral [18], antithrombotic [19], antibactericidal [20], antituberculosis [21], and anticancer activity [22].

In this article, the characteristics and properties that enable the calixarenes to self-assemble in order to form amphiphilic assemblies, micelles of cylindrical or ellipsoidal geometry, vesicles, bilayers, liposomes, and nanoparticles such as nanocapsules and nanospheres that allow the encapsulation, transport, and controlled release of drugs or that can play an important role in the stability, bioavailability, solubility, or even the biological activity of the drugs are reviewed. Also reviewed are the types of molecules that can be recognized by calixarenes, their interactions, and the techniques that allow their determination and the evaluation of the stoichiometry of the complex formed in solution and solid state. In particular, calixarene toxicity, their characteristics, the variation in the size of the cavity and in their functional groups, and their applications, as well as the advantages of these macrocycles, are described.

## 2. Calix[*n*]arenes

### 2.1. Summary Description of Its Structure

**Figure 1:**
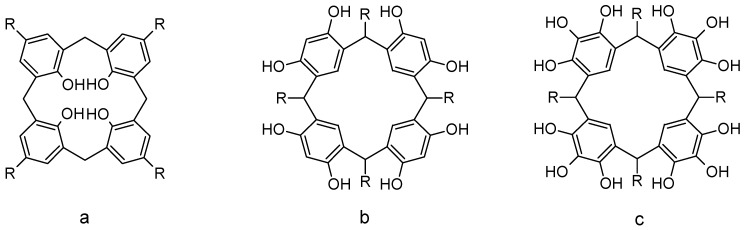
Polyhydroxylated plataforms. (**a**) Calix[4]arene; (**b**) resorcinarene; (**c**) pyrogalloarene.

Polyhydroxylated platforms such as calixarenes, resorcinarenes, or pyrogalloarenes (Figure 1) are a very interesting class of compounds. They have a remarkable ability to act as receptors for a variety of guest species, depending on their structural properties [23,24,25,26,27], which can be modified by changing the size of the substituents or by adding functional groups as a part of the scaffold [28,29,30,31,32]. Among the described macrocyclic compounds, calixarenes are probably the most promising for application in the area of host–guest recognition of toxicological molecules. Their synthetic availability, low toxicity, and presence of reactive sides are characteristics that make them relevant within supramolecular chemistry.

**Figure 2:**
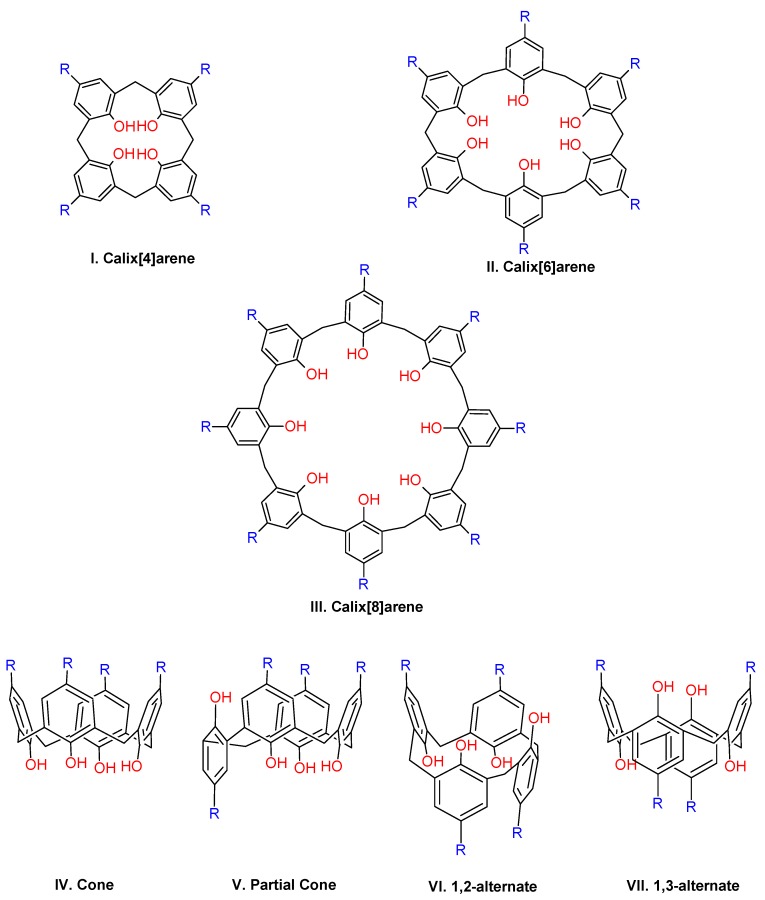
Structures of calix[4]arenes.

The calixarenes are a family of macrocyclic compounds with a variable number of phenol units linked by methylene bridges in *ortho* position [33]. The number of units of aromatics can be between 4 and 20, although the calixarenes of 4, 5, 6, 7, and 8 are the most common [34,35]. As shown in Figure 2, the cyclic structure of calix[*n*]arenes is similar to other polyhydroxylated macrocyles [36,37], and the macrocycle cavity will depend on the number of aromatic units in the system. These compounds have a three-dimensional cavity that can accommodate host molecules during a process called host–guest complexation. These systems have an advantage as a synthetic receptor, owing to different conformational isomeric forms, which allow different uses and applications. For instance, the calix[4]arenes can adopt several different conformers, including the cone, partial cone, 1,2-alternate, and 1,3-alternate. In the rigid cone conformation, all the phenolic –OH groups form strong hydrogen bonds that stabilize the structure (Figure 2).

**Figure 3:**
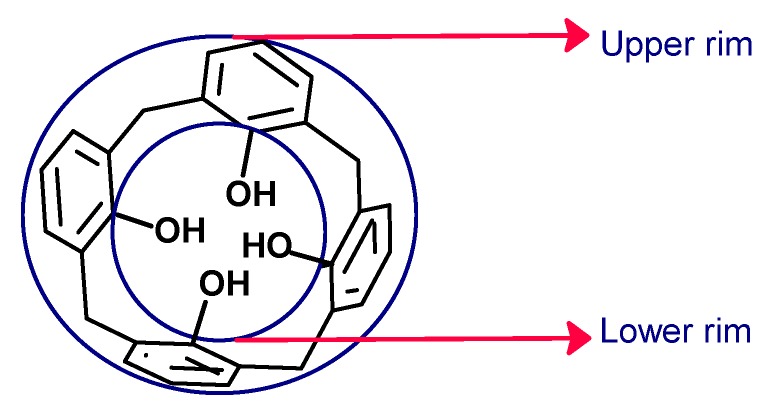
Upper rim and the lower rim on the cavity of the calix[4]arenes.

In the cavity of calix[4]arene in the cone conformation, it is possible to distinguish two edges: the lower rim, where the methylene bridges are, and the upper rim on the opposite side. In the center is the annular system, where the aromatic rings are; in addition, the upper rim is larger than the lower rim (Figure 3) [38]. The molecular dimensions of the cavities vary depending on the number of units of aromatics; thus, the diameter of the upper rim has been estimated to be 3.8 Å for calix[4]arenes and 5.0 Å for calix[6]arenes [39].

**Figure 4:**
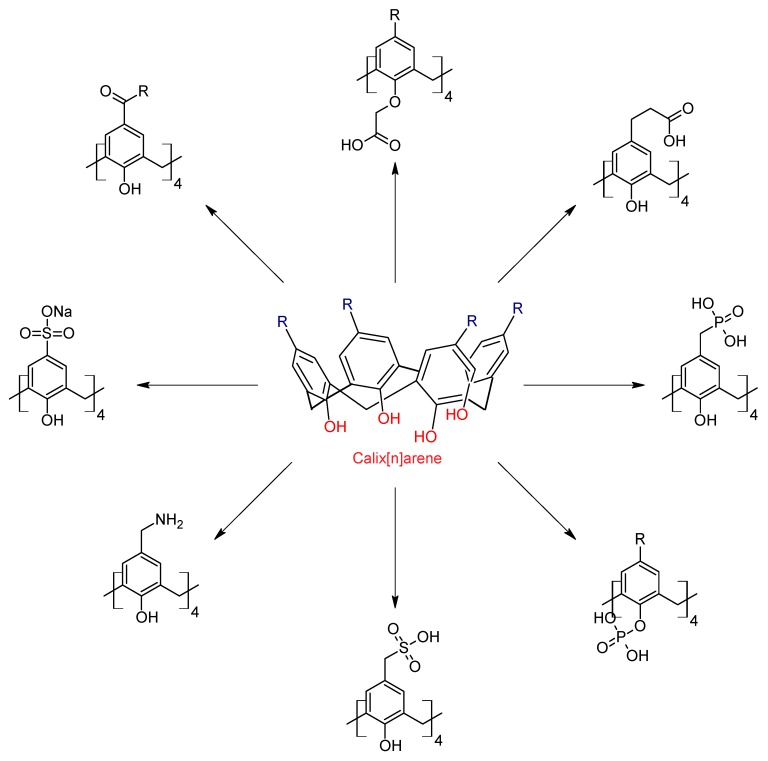
Polar derivatives of calixarenes.

The calix[4]arenes can be functionalized on the upper rim or on the lower rim with several functional groups such as amides, imines, sulfur, azo, semicarbazone, and alkyl groups, among others, producing a wide variety of macrocycle compounds with different properties of recognition, selectivity, solubility, and degree of hydrophobicity [40]. This last aspect is very important, because with the introduction of polar groups to the calixarenes it is possible to design amphiphilic macrocycles with a hydrophilic head and a hydrophobic tail, which may self-assemble into micelles, vesicles, liposomes, and other aggregates useful in the transport of drugs [19]. Functionalization of calixarenes can be done from the starting materials; varying the nature of the substituent group on the phenol facilitates the modification on the upper rim of the macrocyclic system. On the lower rim, the obvious site for chemical modification is that of the hydroxyl groups. The functionalization of calixarenes with polar groups can lead to various structures, as shown in Figure 4. The reactivity of the calixarenes is mainly located at two points: on the hydroxyl groups (lower rim) or on the position of the hydroxyl group (upper rim). Functioning substances such as carboxilates [41], phosphates [42], ammonium groups [43] or sulfonates [44] can be introduced into the hydroxylated platform by means of easily accessible reactions and selectively, with good yields.

The importance of the calix[*n*]arenes lies in the fact that they are able to recognize and accommodate into their cavity guest molecules via non-covalent interactions. Some of these interactions are H-bonding, cation–π, π–π stacking, van der Waals interactions, and the so-called anion–π, in cases where the aromatic system is electron-deficient [45,46]. The magnitude of the interaction also depends on the conformation of the macrocycle [47].

In conclusion, the advantages of the calix[4]arenes are that they are easy to synthetize and that they can be modified to obtain compounds according to the guest that one wants to complex. This synthesis has good yields, and the reagents are inexpensive [48]. In addition, the simple derivatives of calixarenes have not exhibited toxicity or immunogenic properties [19]. All of this makes calixarenes highly valued in supramolecular chemistry as complex agents for the transport of drugs and their controlled release, among other applications.

### 2.2. Complexing Properties of Calixarenes

**Figure 5:**
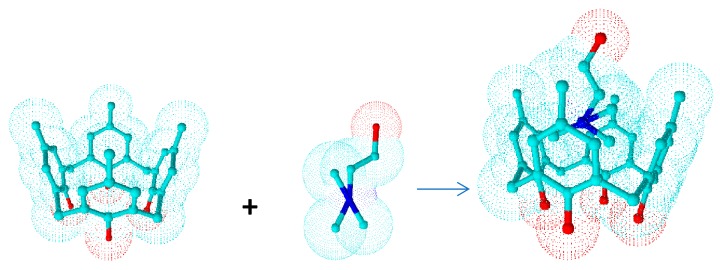
Three-dimensional representation of the complexation process of calix[4]arenes.

As indicated, the cavity of the calixarenes is of variable size and is sufficiently large to accommodate anions, cations, or neutral molecules, including biologically important molecules. This, together with their ability to trap guest molecules by means of noncovalent interactions, has resulted in widespread interest in the calix[*n*]arenes within supramolecular chemistry, particularly with respect to the host–guest phenomena (Figure 5) [49,50].

Furthermore, as mentioned above, the calix[*n*]arenes can be functionalized at both rims, R and –OH (Figure 3) [51,52,53], producing a wide variety of compounds that can have different complexing properties. In particular, calix[*n*]arenes functionalized with a sulfonic group in the para position have been found to have several pharmaceutical applications, due to their potential for encapsulating drugs, increasing their solubility, bioavailability, oral absorption, and stability under heat, light, and acidic conditions [54,55,56]. In addition, they possess good biocompatibility and innocuousness [56,57]. Other applications of the calix[*n*]arenes are as phase-transfer agents, sensors, antibacterials, ion-selective electrodes, and use as catalysts and in separations science [58,59].

Other advantages of the calix[*n*]arenes over other macrocyclic systems include a preorganized cavity of variable size and an electron-rich option of modification and selective binding with the guest [58,59]. This increases their potential use as agents of specific recognition, for example of a guest of toxicological interest.

### 2.3. Amphiphilic Assemblies Based on Calixarenes

**Figure 6:**
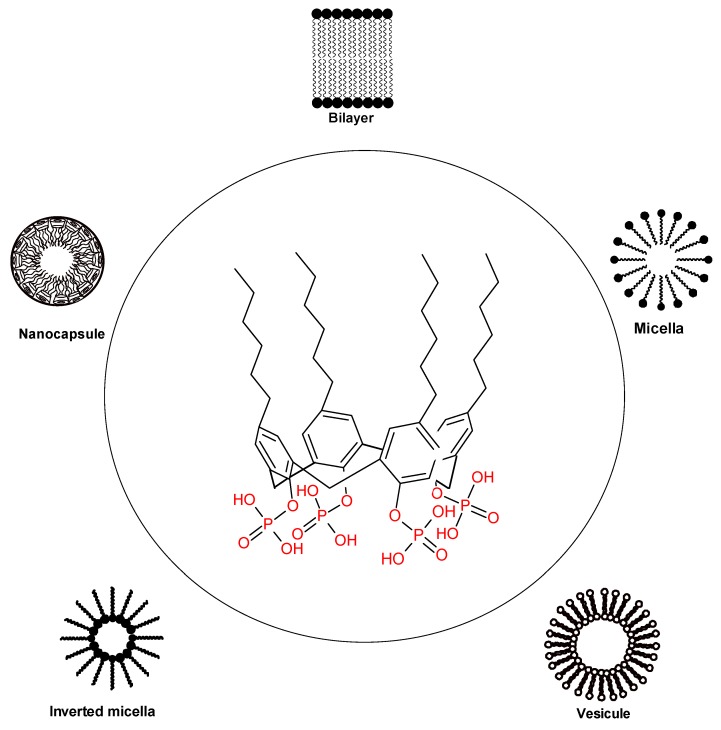
Possibilities of amphiphilic calixarenes in self-assembly.

As was discussed above, calixarenes are substances of easy access and of easy chemical modification, thus allowing one to obtain systems with the introduction of polar groups to the calixarenes, making it possible to design amphiphilic macrocycles with a hydrophilic head and a hydrophobic tail, which may self-assemble into micelles, vesicles, liposomes, nanocapsules, and other aggregates. The development of self-organizing synthetic amphiphilic calixarenes with properties of inclusion and encapsulation allows complex hydrophobic molecules to be transported in a hydrophilic environment; this is especially useful in the transport of drugs (Figure 6).

### 2.4. Types of Molecules that Can Be Recognized by Calixarenes and Their Effect

As mentioned above, the calixarenes have potential applications in diverse fields of medicine and in the pharmaceutical and biological contexts. They are very versatile molecules, because they have a hydrophobic character, but when aqueous environments are needed, for example in biological media, the calixarenes can be modified by the introduction of hydrophilic groups at the upper and lower rim of the macrocycle, leading to water-soluble structures. In this section, we give a brief description of both hydrophilic and hydrophobic molecules that can be recognized or transported by calixarenes.

#### 2.4.1. Recognition of Dopamine by Calixarenes

**Figure 7:**
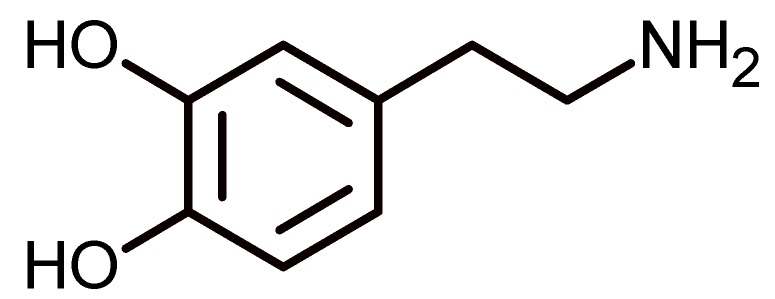
Chemical structure of dopamine.

Among the many substances that can be recognized by calixarenes is dopamine (Figure 7), which is a neurotransmitter of vital importance for the normal function of the central nervous system. The lack of this substance is one of the causes of Parkinson’s disease, an illness that affects more than 10 million people worldwide [60]. In addition, alterations in the normal levels of dopamine are associated with attention-deficit hyperactivity disorder, which affects between 2 and 7% of children, adolescents, and adults in the world [61].

Dopamine can be recognized by 4-*tert*-butylcalix[6]arene when it is co-spread with cellulose acetate to form a Langmuir film and transferred to a gold electrode. The cellulose acetate maintaining the calixarene remains in the vertical cone conformation in the air-water interface, while the presence of calixarene in the Langmuir film provides selectivity to a sensor. The lineal range where the dopamine can be detected is between 5 and 100, and 100 and 7500 nm, and the limit of detection is 2.54 nM. The ratio of calixarene/cellulose acetate has been optimized to show that when the percent of calixarene is 30 wt %, the reuptake of the dopamine is greatest [62].

#### 2.4.2. Improved Solubility of Testosterone with Calixarene

**Figure 8:**
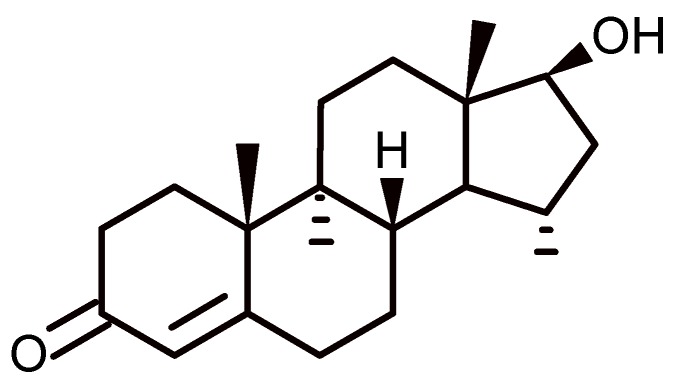
Chemical structure of testosterone.

The interaction of calixarenes with steroids has been studied by several authors. In particular, a study of the complexation of testosterone with a water-soluble calixarene has been published by Millership [63]. Testosterone (Figure 8) is a steroid hormone involved in the male sexual response. It is recognized that erectile dysfunction can result from low levels of testosterone, and therefore the assessment of serum testosterone levels before establishing a possible treatment is important (Figure 2) [64].

Because testosterone is not soluble in water, a method that allows its solubilization is desirable. In this way, Millership improved the solubility of this steroid in water by means of the complexation of the testosterone with 4-sulphonic calix[*n*]arenes. The author measured the solubility in the presence and absence of several calixarenes, such as 4-sulphonic calix[4]arenes, 4-sulphonic calix[6]arenes, and 4-sulphonic calix[8]arenes below the critical micelle concentration, and in all cases found the formation of soluble complexes 1:1, with 4-sulphonic calix[8]arene being the most soluble. This was explained by the fact that the steroid does not enter into the cavity of 4-sulphonic calix[4]arenes, due to their small size, while it can enter comfortably into the cavity of 4-sulphonic calix[6]arenes and 4-sulphonic calix[8]arene. He also found that the pH of the solution influences the shape of the calixarene because of the effect of the hydrogen bonding of the hydroxyl groups. Experiments conducted in phosphate buffers at pH 5.8, 7.3, and 10 demonstrate that the greatest solubility is reached at pH 7.3, which suggests that the conformations adopted by calixarene at this pH favor the process of complexation. The complex constants at pH 7.3 for complexes testosterone–4-sulphonic calix[4]arenes, testosterone–4-sulphonic calix[6]arenes, and testosterone–4-sulphonic calix[8]arenes were 26± 22, 346 ± 39, and 156 ± 9, respectively [65].

#### 2.4.3. Biomaterial Modification with Calixarenes to Avoid Allergy or Infection

**Figure 9:**
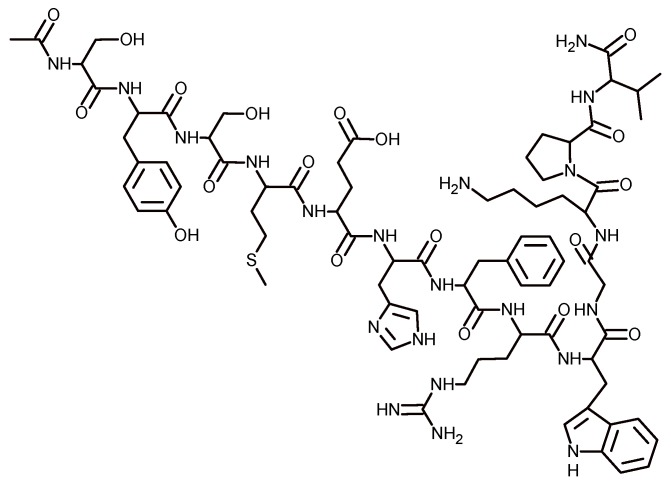
Chemical structure of α-melanocyte hormone.

There are several implantable medical devices useful for the repair of soft and hard tissue, but many of these produce acute inflammation. Charnley et al. [65] proposed coating these medical devices with a natural anti-inflammatory that consists of a hormone produced by the organism that does not generate allergy or infection but that can easily be synthesized in the laboratory. In this study, the hormone was α-melanocyte-stimulating (Figure 9), and was immobilized onto medical device surfaces with *C*-tetra(octyl)calixresorcin[4]arene. The results obtained by the authors indicated that the α-melanocyte hormone retains its anti-inflammatory properties and suggested that this strategy could be useful in the manufacture of new materials [66].

Other applications similar to this one have been reported with proteins, which are important for the manufacture of synthetic organs, drug delivery systems, and biosensing, among other uses. The aim is to achieve an efficient interaction between the proteins and the solid materials for the production of nano- and biomaterials. In this context, the calixarenes have been shown to be useful as linking agents. For example, Keskinates et al. [66] used a calixarene tetraester (5,11,17,23-tetra-*tert*-butyl-25,26,27,28-tetramethoxycarbonylmethoxy-calix[4]arene) to achieve a good interaction between nanofibers of polyacrylonitrile (PAN) or poly(methyl methacrylate) (PMMA) and a green fluorescent protein. The containment of calixarene on the fiber where the binding of protein was highest was 50%. The usefulness of the modified fiber is as an adsorbent membrane for the removal of proteins in aqueous solution; therefore the authors suggest that PAN or PMMA fibers containing calixarene are a promising new and inexpensive material for protein purification [67].

#### 2.4.4. Combination of Calixarenes and Cyclodextrins to Improve the Solubility of an Anthelmintic Drug

**Figure 10:**
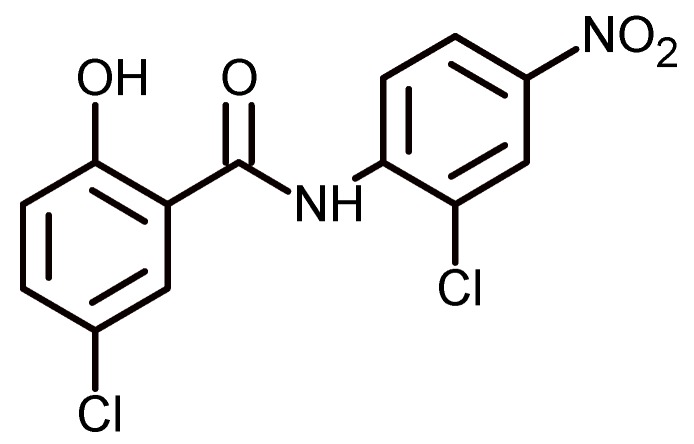
Niclosamide structure, an anthelmintic drug used to treat parasitic infections.

Niclosamide (Figure 10) is an oral anthelmintic drug used worldwide to treat parasitic infections. It is active against beef and dog tapeworms. In addition, it is useful for treating diseases caused by these parasites, such as cancer, metabolic diseases, artery constriction, endometriosis, and rheumatoid arthritis, among others [68]. In spite of its broad clinical application, the efficacy of niclosamide can be affected by its very low solubility in water (230 ng/mL). Studies conducted by Yang et al. have shown a great increase in the aqueous solubility niclosamide by means of complexation with 4-sulphonate-calix[6]arene and hydroxypropyl-β-cyclodextrin. The advantage of the use of water-soluble calixarenes is that they provide a hydrophobic environment to include the drug and a hydrophilic head (sulphonate groups) that allow its solubilization. The combination of the two macrocycle types provides both the properties of cyclodextrins and those of micelles. Another advantage is that the *p*-sulphonate-calix[4, 6, and 8]arenes do not exhibit acute toxication when they are injected into mice, and exhibit no activity in the Ames test. In addition, the inner cavity diameter is variable, between 3.0, 7.6, and 11.7 Å for calix[4]arene, calix[6]arene, and calix[8]arene, respectively, and between 5.7, 7.8, and 9.5 Å for α, β, and γ-cyclodextrins. Furthermore, calixarenes are highly flexible molecules, while the cyclodextrins are quite rigid molecules. All of the above ensures good versatility of the binding of the two macrocycles with the anthelmintic drug.

The authors indicate that there is an increase of solubility of niclosamide with an increase in equal molar concentrations of calixarene and cyclodextrin, but when the concentrations reach 0.005 M, the solubility decreases, indicating the precipitation of an insoluble complex at higher concentrations. The process of complexation was explained by hydrogen bonding between the hydroxylic groups of calixarene and the oxygen atom and the nitrogen atom of niclosamide. Hydrophobic interactions also can be suggested between the hydrophobic cavity of calixarene and the hydrophobic molecule of niclosamide. On the other hand, the solubility of niclosamide also increases with the cyclodextrin, because these molecules establish hydrogen bonding with the hydroxyl groups on the exterior of the cyclodextrin. In general terms, the great increase of solubility of niclosamide is attributed to the additivity of solubility to 4-sulphonato-calix[6]arene and 2-hydroxypropyl-β-cyclodextrin [69].

#### 2.4.5. Drug Delivery Systems Based on Calixarenes

Calixarenes can also be useful as nanocarriers in the form of inclusion complexes, micelles, hydrogels, vesicles, liposomes, and nanoparticles. For example, calixarenes have attracted attention in medicine for the treatment of cancer, because they can respond to multiple stimuli, are stable, can avoid nonspecific cell uptake, and are able to reach the target on tumor sites in order to effect the treatment. Calixarenes are ideal for applications in delivery systems, because they have shown good biocompatibility and non-cytotoxicity.

**Figure 11:**
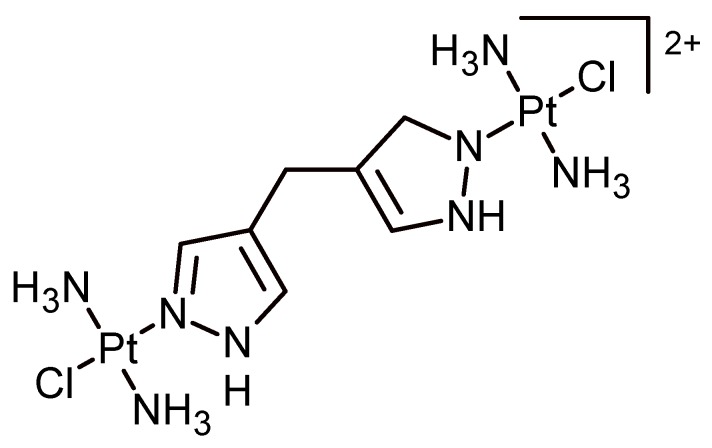
Chemical structure of dinuclear platinum complex trans-[[PtCl(NH_3_)_2_]_2_µ-dpzm]^2+^.

Cisplatin (Figure 11) is an anticancer agent well known for more than 40 years. There are several examples of formulations of oxaliplatin that use liposomes and micelles as a drug delivery vehicle. Abbott et al. published a study of the complexation of a dinuclear platinum complex with *p*-sulphonatocalix[4]arene. The host–guest ratio was 1:1, and the binding constant was 6.8 · 10^4^ M^−1^.

The host–guest complex displays no cytotoxicity and is formed by interactions of hydrogen bonding between the NH_3_ groups of the metal complex with sulphate groups of the calix[4]arene.

#### 2.4.6. Controlled Release of Doxorubicin by Vesicles Based on Calixarenes

**Figure 12:**
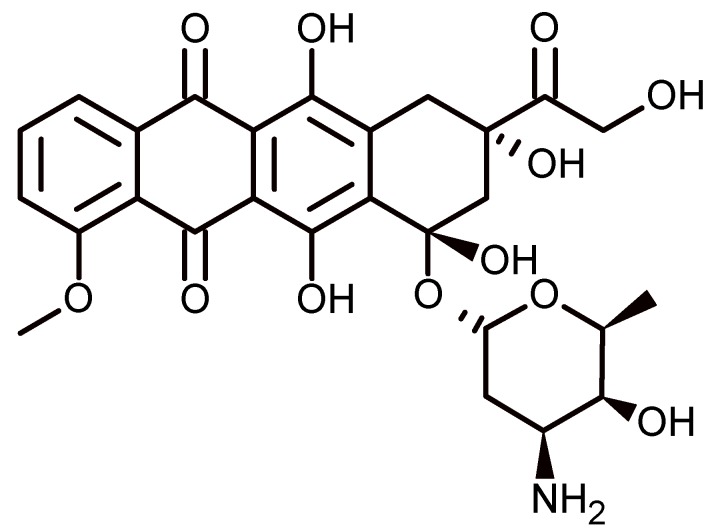
Doxorubicin structure.

Because calixarenes are able to self-assemble, their use for the construction of vesicles is possible. Wang et al. report the construction of binary supramolecular vesicles driven by host–guest complexation between a water-soluble calixarene and viologen. The vesicle obtained can be used as a system of controlled release of doxorubicin hydrochlodire (DOX) (Figure 12), a fluorescent dye that is used in the treatment of cancer. Cell experiments show that the release of DOX by the vesicle does not affect the therapeutic effect of the drug against the cancer cell and by contrast reduces damage to normal cells. The vesicles are stable for 100 h (approximately 4 days) at room temperature. The formation of vesicles was followed by ultraviolet–visible (UV–Vis). In the absence of calixarene, the maximum absorption of the viologen is 260 nm, and the aggregate is not formed, but in the presence of the macrocycle, aggregation takes place through the formation of a complex, and the maximum absorption changes to 450 nm. The stability of the vesicle is reinforced by the electrostatic interaction of the negative groups of sulfonate groups of resorcinarene and the positive groups of viologen.

The obtained vesicles can respond to external stimuli such as temperature, host–guest inclusion, and redox. In the first case, at temperatures between 5 and 70 °C the processes of assembly and disassemblyare produced, respectively. The increasing temperature produced the gradual disassembly of the vesicle. In the second case, taking advantage of the fact that the cyclodextrins can form complexes with viologen, the gradual addition of α-cyclodextrins was carried out, producing the disruption of the vesicle. In the last case, it is known that viologens can be transformed from neutral molecules into the corresponding radical cations by chemical or electrochemical means. The chemical reduction is carried out with hydrazine, where the solution color changes to purple; however, this does not produce the disruption of the vesicle, but the average diameter decreases from 308 to 153 nm. A similar effect is achieved with electrical redox, but when a reduction potential of −1.6 V vs. Ag/AgCl is applied for 30 min, disassembly of the supramolecular vesicle occurs [70].

The above discussion shows that the calixarenes are able to recognize a great diversity of chemical species, depending on the cavity size and the shape and functional groups of the macrocycle. This property is of great importance, because it allows improved bioavailability, solubility, or even activity of molecules of biological interest. In addition, the calixarenes are able to self-assemble to form micelles, vesicles, liposomes, and nanoparticles that allow the encapsulation, transport, and controlled release of drugs. Table 1 shows an overview of the aforementioned applications, where the complexation with calixarene improved the properties of the biomolecule. Also included are methods, conditions, and bibliographic references.

## 3. Conclusions

A wide range of substances of biological interest can be recognized by calix[*n*]arenes. The recognition process depends on the size, shape, polarity, type of functional groups present, aggregation state of the macrocycle, and the formation of non-covalent interactions with the guest. The design of calix[*n*]arenes allows for obtaining amphiphilic molecules able to form higher-order structures such as micelles, vesicles, liposomes, and nanoparticles, which have been shown to be suitable for controlled release drug delivery systems. In addition, calix[4]aneres are useful for improving the solubility, biocompatibility, stability, and bioavailability of biomolecules and molecules with biological activity, and due to the fact that they are not cytotoxic, they can be used in the manufacture of biomaterials. All the above shows the great potential of these macrocycles in pharmacology, biomaterials engineering, and medicine, among other fields.

## Figures and Tables

**Table 1:** Calix[*n*]arenes used for the complexation molecules of biological interest.

Molecule of Biological Interest	Calixarenes Used	Method	Conditions	Ref.
Dopamine	4-*tert*-butylcalix[6]arene	Electrochemical sensing using Langmuir–Blodgett films	The Langmuir film was formed by cellulose acetate and 4-*tert*-butylcalix[6]arene and was transferred to a gold electrode The detection range was 5–100 and 100–7500 nm and the limit of detection was 2.54 nM	[62]
Testosterone	4-sulphonic calix[4]arenes, 4-sulphonic calix[6]arenes, 4-sulphonic calix[8]arenes.	The concentration of testosterone was determined by HPLC method	The solubility of samples were measured at 25 °C at pH 5.8, 7.3, and 10	[63]
α-Melanocyte hormone	*C*-tetra(octyl)calixresorcin [4]arene	The surface was characterized by XPS and MALDI-ToF MS	The peptide was attached to the calixarene with PEG-350 and then coated onto glass	[65]
Green fluorescent protein	5,11,17,23-tetra-*tert*-butyl-25,26,27,28-tetramethoxycarbonylmethoxy-calix[4]arene	The fiber modified was characterized by FTIR, TGA analysis, UV–Vis, fluorescence microscopy and SEM	The bindings studied were done at room temperature The protein content was analysed via UV–Vis at 476 nm The synthesis of calix[4]arene tetra ester derivative was carried out on diphenyl ether with formaldehyde and a basic medium	[67]
Niclosamide (anthelmintic drug)	4-sulphonatocalix[6]arene	The complexation between calixarene-cyclodextrin and niclosamide was followed by thermal analysis. The niclosamide content was determined by HPLC	Solubility studies were done in a pH 7.0 buffer at 30 °C and ionic strength of 0.5 mol/L	[67]
Dinuclear platinum complex	*p*-sulphonatocalix[4]arene	The complex was examined using ^1^H nuclear magnetic resonance and electrospray ionization mass spectrometry, among others	The complex was formed by an equimolar mixture of dinuclear platinum complex with *p*-sulphonatocalix[4]arene Its water solubility is around 4.5 mM	[71]
Doxorubicin	*p*-sulphonatocalix[4]arene	The nanosupramolecular binary vesicles was studied by UV–Vis, fluorescence spectroscopy, dynamic laser scattering, transmission electron microscopy, scanning electron microscopy, and surface tension	The binary vesicles are formed in the presence of asymmetric viologen The maximum absorption of the vesicle was at 450 nm	[70]

FTIR: Fourier-transform infrared; HPLC: High-performance liquid chromatography; MALDI-ToF MS: Matrix-assisted laser desorption/ionization time-of-flight mass spectrometry; SEM: Scanning electron microscopy; TGA: Thermogravimetric analisys; UV–Vis: Ultraviolet—visible; XSP: X-ray photoelectron spectroscopy.
